# Transcriptional analysis of the *Escherichia coli* ColV-Ia plasmid pS88 during growth in human serum and urine

**DOI:** 10.1186/1471-2180-12-115

**Published:** 2012-06-21

**Authors:** Chloé Lemaître, Philippe Bidet, Edouard Bingen, Stéphane Bonacorsi

**Affiliations:** 1 Univ Paris Diderot, Sorbonne Paris Cité, EA 3105, Paris, F-75205, France; 2 AP-HP, Laboratoire de Microbiologie, Hôpital Robert-Debré, Paris, F-75019, France

**Keywords:** *Escherichia coli*, Plasmid, Transcriptome, RNA, Virulence, Iron, Siderophore

## Abstract

**Background:**

The sequenced O45:K1:H7 *Escherichia coli* meningitis strain S88 harbors a large virulence plasmid. To identify possible genetic determinants of pS88 virulence, we examined the transcriptomes of 88 plasmidic ORFs corresponding to known and putative virulence genes, and 35 ORFs of unknown function.

**Results:**

Quantification of plasmidic transcripts was obtained by quantitative real-time reverse transcription of extracted RNA, normalized on three housekeeping genes. The transcriptome of *E. coli* strain S88 grown in human serum and urine e*x vivo* were compared to that obtained during growth in Luria Bertani broth, with and without iron depletion. We also analyzed the transcriptome of a pS88-like plasmid recovered from a neonate with urinary tract infection. The transcriptome obtained after *ex vivo* growth in serum and urine was very similar to those obtained in iron-depleted LB broth. Genes encoding iron acquisition systems were strongly upregulated. *ShiF* and ORF 123, two ORFs encoding protein with hypothetical function and physically linked to aerobactin and salmochelin loci, respectively, were also highly expressed in iron-depleted conditions and may correspond to ancillary iron acquisition genes. Four ORFs were induced *ex vivo*, independently of the iron concentration. Other putative virulence genes such as *iss*, *etsC, ompTp* and *hlyF* were not upregulated in any of the conditions studied. Transcriptome analysis of the pS88-like plasmid recovered *in vivo* showed a similar pattern of induction but at much higher levels.

**Conclusion:**

We identify new pS88 genes potentially involved in the growth of *E. coli* meningitis strain S88 in human serum and urine.

## Background

*Escherichia coli* clone O45:K1:H7, belonging to virulence sequence type (ST)95, is a major cause of neonatal meningitis and of urosepsis in young infants in France [1,2]. The recently sequenced O45:K1:H7 strain S88, isolated from cerebrospinal fluid of a neonate, harbors a plasmid of 134 kb, named pS88, involved in meningeal virulence and bacteremia [3]. Epidemiological studies have shown that major genetic determinants of this plasmid are not restricted to *E. coli* clone O45:K1:H7 but are widely distributed among *E. coli* neonatal meningitis (ECNM) clones, uropathogenic *E. coli* strains (UPEC), and avian pathogenic *E. coli* strains (APEC) [3-6]. Sequencing of pS88 revealed 157 ORFs, including genes involved in the plasmid machinery (transfer, maintenance and replication), IS-like genes, two colicins (colicin Ia and microcin V), and several virulence genes of known or putative functions, such iron-uptake system. These iron-uptake systems include aerobactin (*iucABCD* and *iutA*), salmochelin (*iroBCDEN*) and the SitABCD transport system [7-9]. The S88 plasmid also contains the serum survival gene *iss*[10,11], the *etsABC* genes, encoding a putative type 1 secretion system [4], *ompT*_*p*_, encoding a putative outer-membrane protease differing from the *E. coli* chromosomal *ompT* gene [12] and *hlyF*, encoding a hemolysin [13]. Finally, 35 ORFs have unknown functions and may represent new virulence genes.

Few studies have analyzed the transcriptional profile of human extraintestinal *E. coli* (ExPEC) strains responsible for urinary tract infection [14-17]. To further unravel the role of pS88 in the virulence of clone O45:K1:H7, we analyzed the transcriptional response of plasmid pS88 to growth in urine and serum, representing two steps required for meningeal invasion [18-21]. We also analyzed the transcriptome of a pS88-like plasmid recovered from a neonate with urinary tract infection (UTI).

## Results and discussion

### Validation of transcriptional analysis

**Figure 1:**
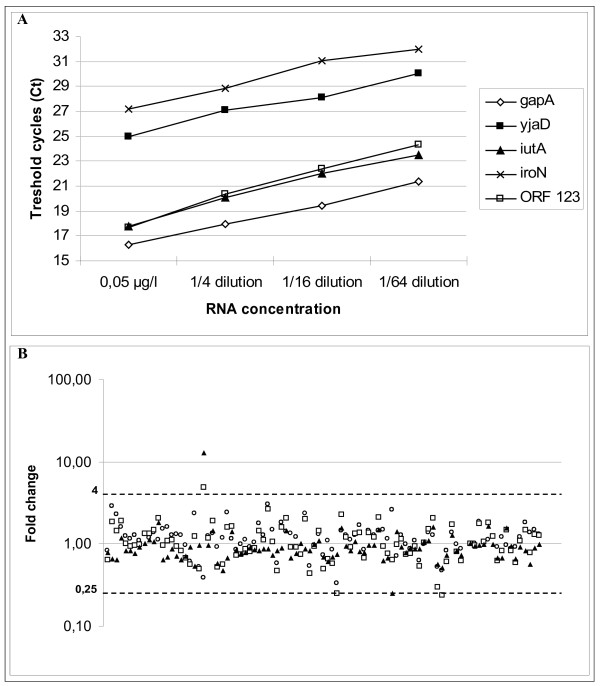
**Linearity and reproducibility of transcriptional analysis.** (**A**) Quantitative RT-PCR of 5 ORFs using different RNA concentrations. (**B**) Analysis of fold changes in RNA transcript abundance by the 2^-ΔΔCT^ method across 3 biological replicates named experiments 1, 2 and 3 after growth in LB broth (experiment 1 vs 2: dots, experiment 1 vs 3: squares, experiment 2 vs 3: triangles). The fold changes fall within the range 0.25-4.00 in 98% of cases.

The transcriptional analysis was validated first by qRT-PCR amplification of transcripts of 5 genes (2 housekeeping genes and 3 plasmidic genes) in serial dilutions of RNA extracted from S88 grown in LB broth. The Ct values showed a linear relation with the template dilution (Figure 1A). Similar results were obtained after growth in LB broth containing the iron chelator 2,2’-dipyridyl (data not shown). We also conduced three independent biological replicates of pS88 after growth in LB Broth, named experiments 1, 2 and 3, to compare the Ct values which each other. As expected, most of the fold changes were close to 1, and 98% of values were between 0.25 and 4 (Figure 1B). Therefore, we considered that an ORF was upregulated or downregulated if the change in expression was smaller or larger than 0.25-fold and 4-fold, respectively, with *p* values ≤0.05. These thresholds are in line with those selected by Mobley *et al.*[16].

### Global analysis of the pS88 transcriptome *ex vivo* and the pAMM transcriptome *in vivo*

Table 1 shows the transcriptome patterns for pS88 grown in iron-depleted LB, in human urine and serum, as well as that of pAMM (recovered from human urine *in vivo*). A transcript was detected for all 88 ORFs tested, except for ORF 23. Overall, 18 ORFs (19%), 10 of which corresponded to 5 operons, were upregulated in at least one of the three *ex vivo* conditions. The only down-regulated genes were *traA* in urine, and *ydfA* and ORF 132 in iron-depleted LB broth. The transcriptome pattern of pAMM largely matched the *ex vivo* patterns, indicating that growth in human urine *ex vivo* was a relevant model. Interestingly, the fold changes observed *in vivo* were far higher than those observed *ex vivo* and *in vitro*.

**Table 1:** Transcriptional expression of pS88 and pAMM ORFs in different growth conditions compared to LB broth

**Name**	**Gene**	**Function**	**LB with iron chelator**^**a**^**pS88**	***p***^**b**^	**Human serum*ex vivo***^**a**^**pS88**	***p***^**b**^	**Human urine*ex vivo***^**a**^**pS88**	***p***^**b**^	**Human urine*in vivo***^**a**^**pAMM**
pS88001	*int*	Putative site-specific recombinase	0.85	0.775	0.59	0.427	0.73	0.505	0.84
pS88002	*repA*	RepFIB replication protein RepA	0.41	0.305	0.97	0.976	0.89	0.889	3.56
pS88003		Conserved hypothetical protein	1.67	0.496	1.26	0.758	3.09	0.159	**7.26**
pS88004		Conserved hypothetical protein	0.93	0.883	0.58	0.266	0.60	0.459	2.52
pS88006		Putative fragment of ImpB UV protection protein	0.48	0.578	0.77	0.550	1.51	0.367	1.17
pS88009	*iutA*	Ferric aerobactin receptor precursor IutA	**4.12**	**0.007**	**4.23**	**0.006**	**4.01**	**0.048**	**9.02**
pS88013	*iucA*	Aerobactin siderophore biosynthesis protein IucA	**45.25**	**0.005**	**15.85**	**0.023**	**18.38**	**0.026**	**168.12**
pS88014	*shiF*	Putative membrane transport protein *ShiF*	**7.66**	**0.006**	**14.03**	**0.005**	**14.19**	**0.004**	**17.71**
pS88015		Putative membrane protein; CrcB-like protein	2.40	0.105	0.82	0.807	**4.19**	0.051	**6.08**
pS88016		Conserved hypothetical protein	1.77	0.250	1.65	0.628	**4.14**	0.066	**9.74**
pS88017		Putative enolase	1.47	0.573	**5.44**	0.152	**7.98**	**0.040**	**18.68**
pS88019	*sitD*	SitD protein; iron transport protein	**4.54**	**0.020**	**38.23**	**0.003**	**26.29**	**0.004**	**139.75**
pS88022	*sitA*	SitA protein; iron transport protein	**17.79**	**0.002**	**49.52**	**0.003**	**83.87**	**0.001**	**776.05**
pS88026		Hypothetical protein	1.32	0.633	1.04	0.959	1.02	0.981	*/*^*c*^
pS88027		Hypothetical protein; putative exported protein	0.70	0.626	1.04	0.956	0.31	0.187	*/*
pS88028		Conserved hypothetical protein	1.11	0.809	0.75	0.577	1.16	0.762	*/*
pS88029		Conserved hypothetical protein	1.30	0.712	1.22	0.751	2.20	0.260	*/*
pS88030		Conserved hypothetical protein	0.30	0.098	0.46	0.308	0.32	0.143	1.09
pS88031		Hypothetical protein	0.67	0.405	0.97	0.959	1.58	0.369	2.08
pS88037	*sopA*	SopA protein (Plasmid partition protein A)	0.60	0.227	0.47	0.147	1.12	0.847	0.98
pS88038	*sopB*	SopB protein (Plasmid partition protein B)	0.38	**0.021**	0.91	0.879	1.41	0.696	3.32
pS88039		Hypothetical protein	0.63	0.312	2.19	0.330	3.82	**0.031**	2.96
pS88040		Conserved hypothetical protein	0.73	0.510	2.74	0.240	3.61	**0.031**	3.61
pS88041		Hypothetical protein	1.39	0.295	0.42	0.174	1.77	0.092	1.47
pS88043		Hypothetical protein	0.89	0.782	1.47	0.378	2.00	0.188	1.83
pS88044	*yubI*	Putative antirestriction protein	1.35	0.720	1.13	0.890	0.99	0.991	3.38
pS88045		Conserved hypothetical protein	0.95	0.919	1.66	0.403	1.09	0.873	**4.52**
pS88046		Conserved hypothetical protein	0.80	0.717	1.38	0.661	1.25	0.735	2.07
pS88047	*ydbA*	Conserved hypothetical protein	1.71	0.542	0.99	0.987	1.33	0.739	**4.18**
pS88048	*ydcA*	Putative adenine-specific DNA methylase	1.44	0.652	1.09	0.917	1.52	0.606	3.98
pS88050	*ssb*	Single-stranded DNA-binding protein	1.56	0.383	2.42	0.152	1.96	0.211	2.91
pS88051	*yubL*	Conserved hypothetical protein	0.90	0.832	1.21	0.842	2.13	0.203	2.05
pS88054	*ycjA*	Putative DNA-binding protein involved in plasmid partitioning (ParB-like partition protein)	1.31	0.260	2.60	0.392	3.45	**0.007**	2.30
pS88055	*psiB*	Plasmid SOS inhibition protein B	0.74	0.414	**5.34**	0.094	3.26	**0.026**	**4.03**
pS88056	*psiA*	Plasmid SOS inhibition protein A	1.67	0.321	**13.06**	**0.048**	**6.44**	**0.016**	3.02
pS88057	*flmC*	Putative F-plasmid maintenance protein C	2.27	0.144	0.55	0.346	0.65	0.401	2.21
pS88059	*yubN*	Conserved hypothetical protein	2.01	0.441	0.90	0.902	1.20	0.826	3.52
pS88060	*yubO*	Conserved hypothetical protein	1.13	0.781	1.79	0.211	2.24	0.075	3.89
pS88061	*yubP*	Conserved hypothetical protein	1.43	0.397	2.40	0.109	1.72	0.408	**4.27**
pS88062	*yubQ*	X polypeptide (P19 protein); Putative transglycosylase	0.94	0.948	0.88	0.910	1.20	0.852	**4.90**
pS88063	*traM*	Protein TraM (Conjugal transfer protein M)	0.77	0.313	0.94	0.866	0.92	0.769	0.25
pS88064	*traJ*	Protein TraJ (Positive regulator of conjugal transfer operon)	0.39	0.212	2.86	0.310	1.08	0.898	1.98
pS88066	*traA*	Fimbrial protein precursor TraA (Pilin)	1.59	0.053	0.54	0.188	0.19	**0.004**	0.21
pS88092	*traT*	Complement resistance and surface exclusion outer membrane protein TraT	0.27	0.265	0.54	0.573	0.82	0.847	0.35
pS88095	*traX*	F pilin acetylase TraX	0.56	0.157	0.54	0.409	0.72	0.389	0.88
pS88096	*finO*	Fertility inhibition protein FinO (Conjugal transfer repressor)	0.49	0.127	0.98	0.968	0.88	0.732	1.21
pS88097	*yigA*	Conserved hypothetical protein YigA	1.22	0.803	2.08	0.427	0.95	0.953	0.50
pS88098	*yigB*	Putative nuclease YigB	0.46	0.241	0.47	0.463	1.34	0.648	2.34
pS88099	*repA2*	Replication regulatory protein RepA2 (Protein CopB)	1.27	0.340	1.43	0.199	2.24	0.071	1.93
pS88100	*repA1*	Replication initiation protein RepA1	0.56	0.120	1.14	0.702	2.18	0.072	1.53
pS88101	*yacA*	Conserved hypothetical protein YacA. possible repressor	0.49	0.344	0.96	0.961	0.41	0.293	**4.30**
pS88102	*yacB*	Putative plasmid stabilization system protein YacB	0.31	0.169	0.64	0.502	0.32	0.227	1.57
pS88103	*yacC*	Putative exoribonuclease YacC	0.38	0.209	0.56	0.461	0.50	0.369	0.95
pS88104	*cia*	Colicin-Ia	**5.11**	0.105	**21.06**	**0.023**	**6.03**	0.087	**70.36**
pS88105	*imm*	Colicin-Ia immunity protein	1.10	0.944	**5.58**	**0.048**	3.46	0.106	3.17
pS88106	*ybaA*	Conserved hypothetical protein YbaA	**5.25**	0.197	**4.87**	0.189	**8.90**	0.096	3.27
pS88108	*ydeA*	Conserved hypothetical protein YdeA	0.45	0.247	0.31	0.165	0.41	0.222	0.51
pS88109	*ydfA*	Conserved hypothetical protein YdfA	0.17	0.119	0.69	0.733	0.36	0.284	0.58
pS88110		Putative acetyltransferase	0.71	0.606	0.98	0.983	0.77	0.684	1.57
pS88111		Predicted dehydrogenase	1.41	0.562	0.31	0.126	0.88	0.801	1.48
pS88112		Predicted dehydrogenase	1.25	0.691	0.63	0.416	1.19	0.736	0.87
pS88113		Predicted dehydrogenase	0.92	0.893	1.13	0.850	1.65	0.509	3.02
pS88114	*cvi*	Microcin V immunity protein	0.84	0.735	1.13	0.846	2.17	0.203	**4.48**
pS88115	*cvaC*	Microcin V precursor (Microcin V bacteriocin)	**21.96**	**0.007**	**17.27**	**0.010**	**29.58**	**0.016**	**61.11**
pS88116	*cvaB*	Microcin V secretion/processing ATP-binding protein CvaB	**12.88**	**0.010**	**17.55**	**0.001**	**19.43**	**0.006**	**162.02**
pS88117	*cvaA*	Microcin V secretion protein CvaA	**26.23**	**0.012**	**44.02**	**0.005**	**43.81**	**0.019**	**215.77**
pS88118		Conserved hypothetical protein	3.99	0.095	**4.66**	0.066	3.32	0.219	**7.46**
pS88123		Putative Phospho-2-dehydro-3-deoxyheptonatealdolase	**354.6**	**0.000**	**190.9**	**0.001**	**109.6**	**0.006**	**144.67**
pS88124	*iroN*	IroN. Salmochelin siderophore receptor	2.94	0.137	2.14	0.465	1.95	0.394	**28.97**
pS88128	*iroB*	IroB. Putative glucosyltransferase	**72.17**	**0.001**	**48.95**	**0.002**	**37.97**	**0.014**	**69.71**
pS88130		Conserved hypothetical protein	1.84	0.336	3.36	0.198	**10.36**	**0.029**	3.10
pS88131		Conserved hypothetical protein	2.43	0.318	**9.11**	**0.031**	**13.83**	**0.039**	**14.66**
pS88132		Hypothetical protein	0.20	**0.013**	0.95	0.871	0.63	0.482	0.40
pS88133	*iss*	Iss (Increased serum survival)	0.28	0.083	0.48	0.282	0.36	0.151	0.66
pS88136		Hypothetical protein	0.93	0.896	1.51	0.618	1.71	0.391	0.65
pS88137		Conserved hypothetical protein; Putative GTPase	0.40	0.263	0.52	0.504	0.64	0.580	1.59
pS88142		Conserved hypothetical protein	0.51	0.096	0.48	0.134	0.77	0.458	*/*
pS88143		Conserved hypothetical protein	0.57	0.090	0.70	0.646	0.84	0.750	*/*
pS88146	*etsC*	Putative type I secretion outer membrane protein EtsC	1.05	0.893	0.42	0.208	0.78	0.478	0.61
pS88148	*etsA*	Putative type I secretion membrane-fusion protein EtsA	0.49	0.126	0.34	0.211	0.36	0.050	0.31
pS88154		Hypothetical protein	0.47	0.330	**4.44**	0.163	1.25	0.790	3.00
pS88155	*ompT*	Outer membrane protease (omptin)	0.48	0.178	0.43	0.092	0.42	0.137	0.37
pS88156	*hlyF*	Hemolysin HlyF	1.02	0.981	0.44	0.402	0.72	0.507	0.14
pS88157		Conserved hypothetical protein; putative Mig-14 protein	1.11	0.921	0.47	0.376	0.94	0.942	0.11
S88-1832	*gapA*^*d*^	Glyceraldehyde-3-phosphate dehydrogenase	1.70	0.396	0.46	0.254	1.15	0.789	0.90
S88-0266	*dinB*^*d*^	DNA polymerase IV	0.69	0.343	2.36	0.131	0.69	0.317	0.90
S88-4457	*yjaD*^*d*^	NADH pyrophophatase	0.85	0.586	0.91	0.698	1.26	0.344	1.24

^*a*^ Fold changes of transcription levels relative to reference condition (growth in LB). Fold change > 4 are in bold print.

^*b*^ p value in Student’s t test for the comparison of the three biological replicates for each experiment in different growth conditions and the reference condition. p < 0.05 are in bold print.

^*c*^ ORFs present in plasmid pS88 but absent from plasmid pAMM. ^*d*^ Housekeeping genes.

### Expression of iron uptake systems

The concentration of free iron in human urine and serum is low, because iron is sequestered by host molecules [22-24]. *E. coli* has developed several strategies to acquire iron in such environments. Ten ORFs were upregulated after growth in urine, in serum, and in iron-depleted LB, suggesting they were induced by the low iron concentrations in these media. Five of these 10 ORFs corresponded to iron-uptake and iron-assimilation systems, namely *iutA* and *iucA* (aerobactin), *iroB* (salmochelin) and *sitA* and *sitB* (SitABCD iron transport system). These iron-uptake systems have previously been linked to the virulence of ExPEC and APEC [4,7-9,24-27]. Mobley *et al.* also observed upregulation of UPEC iron-acquisition systems such as aerobactin, salmochelin and the SitABCD system in urinary isolates from experimentally infected mice and from women with UTI [14,16]. Likewise, Li *et al.* found that genes involved in iron acquisition were among the most significantly upregulated genes during growth in chicken serum of the APEC strain O1 [28], which harbours a plasmid (pAPEC-O1-ColBM) closely related to pS88 [3]. Our study represents the first transcriptional analysis of an *E. coli* plasmid after growth in human serum.

Surprisingly, we found that the salmochelin receptor *iroN* was not upregulated in our *ex vivo* experiments, and that the transcript level of the aerobactin receptor *iutA* was markedly lower than that of the siderophore *iucA.* In contrast the salmochelin receptor *iroN* was upregulated 28-fold in the isolate from a neonate with UTI. Such discrepancies have been previously described. In the murine UTI model used by Mobley *et al.*[16], *iroN* was upregulated but its transcript level was also lower than that of *iroB*. Moreover, in their transcriptome analysis of *E. coli* isolates from eight women with urinary tract infection, *iroN* and *iutA* were only upregulated in two isolates [14].

### Colicin expression

Another group of genes upregulated in iron-deficient conditions were the genes encoding the Microcin V (*cvaAcvaBcvaC*) and Colicin Ia, which were also upregulated in human serum and urine. Previous reports have shown the influence of bacterial intracellular iron levels on colicin expression, but the reason of such induction is still poorly understood [29-31]. Of note, transcription of immunity protein for both colicins was not upregulated in any of the conditions studied except for Colicin Ia in human serum.

### Expression of ORFs of unknown function in iron-deficient environments

Two ORFs with unknown functions, *shiF* and ORF 123, were upregulated in iron-deficient conditions, with large fold changes *in vivo* and *ex vivo*.

ORF 123 was the most strongly upregulated (> 100-fold) in the 3 test conditions, and was expressed 3 to 4 times more strongly than the iron acquisition systems. A nucleotide homology search using the BLAST program [32] showed that ORF 123 is highly homologous (99%) to an ORF present in *E. coli* plasmids possessing a CVP region (such pAPEC-O1-ColI-BM, pAPEC-O2-ColV and pAPEC-1) or located on the chromosome of UPEC strains such as CFT073 (ORF c1220; 94%) and 536 (ORF ECP–0281; 95%). No homologous gene is found in the commensal *E. coli* strain MG1655. Transcriptome analysis by Mobley *et al.*[16] showed over-expression of c1220 transcripts in *E. coli* CFT073 in a mouse model of UTI. The putative protein encoded by ORF 123 showed 45-50% identity to three phospho-2-dehydro-3-deoxyheptonate aldolases that catalyze the first reaction of the shikimate pathway and are present on the chromosome of *E. coli* K12. This pathway involves seven enzymatic reactions that generate chorismate, a factor involved in the synthesis of three aromatic amino acids (tyrosine, tryptophan and phenylalanine) [33]. However, this pathway is also involved in other reactions, such as biosynthesis of siderophore group nonribosomal peptides such as yersiniabactin and enterobactin. In plasmid pS88, as in other CVP-containing plasmids, ORF 123 lies just upstream of *iroN* and is preceded by a sequence resembling the Fur Box consensus sequence (5'-GATAATGATAATCATTATC) [34,35]. BLAST analysis of complete genomes available on publicly available database showed that ORF 123 is only found when the salmochelin operon is present but the reciprocity is not true, as for example in strain UTI89, which harbors only an *iro* locus. On the chromosome of *E. coli* strains CFT073 and 536, this ORF (c1220 and ECP_0281, respectively) is located in a pathogenicity island containing an *iro* locus but is 20–30 kb distant from the *iro* locus. Because of its putative function, its high inducibility in iron-depleted conditions, and its physical proximity to the *iro* locus, we suspected that this ORF might be an auxiliary gene that boosts the synthesis of iron acquisition systems such as salmochelin by enhancing the production of chorismate and, consequently, enterobactin, the precursor of salmochelin [36].

The *shiF* ORF was also upregulated in iron-deficient environments. *ShiF* was first described in the pathogenicity island SHI-2 in *Shigella flexneri*[37] and encodes a putative protein belonging to the major facilitator superfamily. The latter is one of the two largest families of membrane transporters capable of transporting small solutes in response to chemiosmotic ion gradients. Transcriptome analysis of APEC O1 grown in chicken serum showed that *shiF* was also upregulated [28]. BLAST analysis revealed that *shiF* is present in many UPEC and APEC strains, but only when the locus encoding aerobactin is present, although the two do not always colocalize. Of interest, in pS88, as in Shi-2, *shiF* is located just upstream of the aerobactin operon, on the opposite strand, and shares the same Fur Box. These results suggest that *shiF* induction is at least partly regulated by iron deficiency and that, like ORF 123, *shiF* may be an auxiliary gene that promotes the transport of lysine, the precursor of aerobactin.

### Specific ORF expression in serum and urine

A minority of ORFs were upregulated in serum and/or urine but not in iron-depleted LB broth. Two of these ORFs were upregulated only in urine (ORFs 17 and 130), while 2 ORFs were upregulated in both serum and urine (*psiA* and ORF 131). Meanwhile the putative role of ORF 130, ORF 131 and *psiA* in the steps studied could not be predicted, the most strongly upregulated ORF in urine, ORF 17, could play a role in the infection process. This ORF codes for a putative enolase, an enzyme involved in the penultimate step of glycolysis and that catalyses 2-phosphoglycerate conversion to phosphoenolpyruvate. Intriguingly this latter molecule is the substrate of the phospho-2-dehydro-3-deoxyheptonate aldolase involved in the shikimate pathway. ORF 17 might therefore help to optimize the synthesis of iron-uptake systems in urine.

### Other putative virulence genes

Other putative virulence factors like *ompTp, etsCiss* and *hlyF*[10-13,38,39] were not upregulated in any of the conditions studied here. Nolan *et al.* has reported upregulation of the *etsABC* genes (but not *iss*) in APEC O1 strains, including pAPEC-O1-ColBM, grown in chicken serum at 37°C [28]. In contrast, in their transcriptional analysis of 8 genes in pAPEC-O2-ColV grown in chicken serum and human urine, they found that *iss*, but not *etsC,* was upregulated in chicken serum [40]. Moreover, *hlyF* was also upregulated in chicken serum but not in human urine. Variability between commercial chicken serum could explain the observed differences in the previously mentioned studies. Alternatively, these putative virulence genes may be induced in highly specific conditions that remain to be determined.

## Conclusion

While several studies have examined *E. coli* virulence gene expression in animal models, little is known about their expression during human infection [14,15]. Here we identified several genes that may play a key role during *E. coli* growth in human serum and urine. Further studies are necessary to determine the roles of these candidate virulence genes and to understand the contribution of plasmid pS88 to the virulence of *E. coli* strain S88, in particular its aptitude to cross the human blood–brain barrier.

## Methods

### Bacteria

*E. coli* meningitis strain S88, representative of the French clonal group O45:K1:H7, has been shown to harbor a virulence plasmid of 134 kb, designated pS88 [3]. *E. coli* strains responsible for UTI in young infants were screened for transcriptional analysis *in vivo*, as follows. The O45-specific genes and K1 capsular antigen were detected as described elsewhere [41,42]. The presence of *issetscChlyF, ompT*_*p*_ and *cvaA*, together with the genes encoding salmochelin (*iroN*), aerobactin (*iucC*) and the iron-uptake system SitABCD (*sitA*), considered to be a signature of a conserved virulence plasmidic (CVP) region characteristic of pS88 [38], were sought by PCR as previously described [3].

### Growth conditions

An overnight culture of strain S88 in Luria Bertani (LB) broth (Sigma) was diluted 1/100 in LB broth and grown at 37°C with agitation until optical density at 600 nm (OD_600_) reached 0.65. This culture represented the reference condition for this study. Strain S88 was also grown in LB broth containing the iron chelator 2,2’-dipyridyl (Sigma, Saint Quentin Fallavier, France) at a final concentration of 200 μM, as previously described [43]. With their informed consent, serum was collected at Etablissement Français du Sang from healthy blood donors aged from 20 to 40 years who had no history of infection or antibiotic use in the previous 2 months. Serums from 20 donors were pooled and aliquots of 500 μl were stored at −80°C until use. Transcriptome analysis of *E. coli* cultured in serum was performed as follows: an overnight culture of S88 in LB broth was diluted 1/10 in physiological saline, then 250 μl of this dilution was mixed with 250 μl of serum and incubated at 37°C for 3 hours; the culture was centrifuged for 7 min at 9000 *g* and 21°C in a microcentrifuge (Jouan) and the pellet was resuspended in 500 μl of physiological saline. RNA was immediately stabilized with RNA Protect Bacterial Reagent (QIAGEN) and the sample was stored at −20°C until RNA extraction. With their parents’ informed consent, sterile urine was collected from healthy children aged from 3 months to 5 years who had no history of UTI or antibiotic use in the previous 2 months, and was stored in aliquots of 5 ml at −20°C. An overnight culture of S88 in LB broth was diluted 1/100 in the pooled urine and cultured at 37°C until OD_600_ reached 0.25 (preliminary experiments showed that this represented the mid-exponential phase of growth in urine). RNA was then stabilized as described above. To analyze the regulation of pS88 expression *in vivo*, aliquots of fresh urine were obtained from children aged no more than 3 months who had been admitted to Robert-Debré Hospital for fever and whose urine contained ≥ 10^6^ leukocytes per ml and numerous Gram-negative rods. The samples were immediately treated with RNA Protect Bacterial Reagent (QIAGEN) and stored at −20°C until RNA extraction. If urine culture yielded ≥10^5^*E. coli* CFU/ml and no other bacteria, confirming the diagnosis of UTI, the serotype was determined and genes characteristic of the CVP region were sought as described above. Among the 10 isolates analyzed, one, designated AMM, was recovered in 2010 from urine of a 2-month-old infant with acute pyelonephritis and no medical history. This strain, belonged to ST95, was of serogroup O45:K1 and harbored the main chromosomal virulence genes (*fuyApapCpapGII*) and the CVP region, indicating that AMM belongs to the O45:K1 clonal group and is very similar to S88. PCRs specific for 88 plasmidic ORFs of interest (see below) showed that the pAMM plasmid possessed 82 of these ORFs. RNA was extracted as described above, directly from urine stored at −20°C, and after growth in LB (reference condition).

### RNA extraction

RNA from *ex vivo* and *in vivo* samples was extracted with the RNeasy Mini kit (QIAGEN) according to the manufacturer’s instructions. Total RNA was then isolated with the RNase-Free DNase set (QIAGEN). The concentration of total RNA was determined with ND-1000 spectrophotometer (NanoDrop) and adjusted to a final concentration of 0.05 μg/μl.

### Quantitative reverse transcription-PCR (qRT-PCR)

For transcriptome analysis, all ORFs of unknown function and between 1 and 4 ORFs with known functions at each plasmid locus except most genes corresponding to plasmid transfer systems, insertion sequences and transposases were chosen. A total of 88 plasmid transcripts were retained for investigation. As previously recommended [44], three housekeeping genes were used for normalization, chosen among previously described genes (*gapAdinB* and *yjaD*) [16,45]. Primers were designed with Primer 3 software [46]. Assays were performed in microplates (Eurogentec), the primer pairs being distributed directly at a concentration of 200 nM with a Eurogentec device. Reverse transcriptase (EuroScript RT, 0.125 U/μl) and RNA extract (0.05 μg/μl) were added to the One-step MESA GREEN qRT-PCR MasterMix Plus for SYBR assay (Eurogentec) according to the manufacturer’s instructions, and the mix was distributed in the microplates (0.05 μg of RNA in each final reaction mix). Reverse transcription and amplification were performed with an LC480 Light Cycler (Roche) in one step with the following cycling parameters: 30 min at 48°C for reverse transcription, 5 min at 95°C for reverse transcriptase inactivation and Taq activation, and 45 cycles of 15 s at 95°C, 20 s at 60°C and 40 s at 72°C. Melting curve analysis of each reaction product was used to control the specificity of qRT-PCR.

### Data and statistical analysis

The cycle threshold (Ct) was automatically determined by using the Second Derivative Maximum Method included in LC480 software. The Ct values provided by the qRT-PCR instrument were imported into a spreadsheet program (Microsoft Excel). The fold change in the abundance of the 88 ORF transcripts between each test condition (growth in LB with 2,2’-dipyridyl, serum and urine) and the reference condition (growth in LB) was calculated by using the 2^-ΔΔCT^ method [47,48]. The average of 3 housekeeping genes (*gapAdinByjaD*) was used for the normalization [44]. Briefly, the first ΔCt represents the difference of Ct between the investigated gene and the average of the 3 housekeeping genes and the ΔΔCt is then calculated using the formula ΔΔCt=ΔCt(test condition)- ΔCt(reference condition). For transcriptome analysis during growth *in vitro* and *ex vivo*, three independent experiments (biological and technical replicates) were performed in each condition, including growth, RNA extraction and qRT-PCR. The *in vivo* experiment was performed only once because of the limited available amount of urine.

A *p* value for each ORF was calculated by using Student’s *t* test to compare the three replicates for each bacterial growth condition.

## Competing interest

All authors declare no financial competing interests.

## Authors contributions

CL carried out all transcriptomic studies and participated in study design. SB and PB conceived of the study, and participated in its design and coordination and wrote the manuscript. EB participated in study design and helped to draft the manuscript. All authors read and approved the final manuscript.
